# Advanced quantitative magnetic resonance imaging of lower extremity muscle microtrauma after marathon: a mini review

**DOI:** 10.3389/fspor.2024.1481731

**Published:** 2024-10-29

**Authors:** Yu Cheng, Xiaokai Li

**Affiliations:** School of Sports and Health, Shanghai University of Sport, Shanghai, China

**Keywords:** magnetic resonance imaging, marathon, muscle, microtrauma, lower extremity

## Abstract

This article reviews the existing literature and outlines recent advances in quantitative Magnetic Resonance Imaging (MRI) techniques for the assessment of lower extremity muscle microtrauma following a marathon. Single-modality quantitative MRI techniques include T2 mapping to assess the dynamics of muscle inflammatory edema and variability at the site of injury, Diffusion Tensor Imaging (DTI) to detect subclinical changes in muscle injury, Intravoxel Incoherent Motion (IVIM) imaging to provide simultaneous information on perfusion and diffusion in muscle tissue without the need for intravenous contrast, and Magnetic Resonance Spectroscopy (MRS) to noninvasively detect intramyocellular lipid (IMCL) content in muscle before and after marathon exercise to explain the use of fatty acids as an energy source in skeletal muscle during long-distance running. As well as Chemical Exchange Saturation Transfer (CEST) is particularly suitable for detecting changes in free creatine, pH values and lactate concentrations in muscles before and after exercise, providing a more detailed picture of muscle physiology and chemistry. These metabolic MRI methods enhance the understanding of biochemical alterations occurring in muscles pre- and post-exercise. Multimodal techniques combine different modalities to provide a comprehensive evaluation of muscle structural and functional changes. These advanced techniques aim to better assess microtrauma and guide clinical treatment, though further validation with larger studies is needed to establish their potential over traditional qualitative methods.

## 1 Introduction

Marathon is a popular pastime and sporting activity, with over 30 million people participating annually across the globe, many of whom are novice runners (1). However, prolonged running (e.g., full marathons, mountain ultra-marathons) tends to lead to organism fatigue and induces motor microtrauma in the muscles of the lower limbs, with common microstructural changes including cellular swelling, loss of membrane integrity, inflammation and fiber tearing (2–5). Although traditional imaging techniques such as Ultrasonography and x-ray offer some advantages in the assessment of sports injuries, with Ultrasonography allowing real-time visualization of soft tissues and x-rays being widely used in skeletal assessment, they have limitations in detecting deeper microstructural changes in muscle and early injury. In contrast, Quantitative Magnetic Resonance Imaging (QMRI) provides higher soft tissue contrast and sensitivity, enabling the assessment of microstructural physiological and biochemical changes in lower limb muscle injuries, which are often not detectable by conventional imaging techniques (6, 7). Whereas detecting changes at the microscopic molecular level in lower limb muscles may be useful for injury prevention, injury assessment in athletes or active individuals, while minimizing injury risk and rehabilitation time (8, 9). Currently, researchers (10–12) are using advanced quantitative imaging techniques to characterize ultramicro changes, monitor recovery, and estimate return-to-exercise time in lower extremity muscle kinesiology injuries in order to avoid gross anatomical damage due to repetitive microtrauma. Therefore, this article focuses on outlining recent advances in advanced quantitative magnetic resonance imaging techniques in assessing lower extremity muscle microtrauma after marathon exercise and directions for future research.

## 2 Materials and methods

A comprehensive literature search was conducted using computerized systems in PubMed, Google Scholar, and China National Knowledge Infrastructure (CNKI) databases. The search utilized specific keywords pertinent to the research questions, as outlined in Table 1. This search encompassed both Chinese and English literature published from 1998 to 2024. Initially, a total of 5,862 articles were identified.

**Table 1:** Literature search keywords.

Concepts (AND)	Magnetic resonance imaging	Marathon	Muscle	Microtrauma
Keywords (OR)	Magnetic resonance imaging	Marathon running	Muscles	Wounds and injuries
			Muscle	Injury
				Microtrauma
	MRI	Marathon	Muscle, skeletal	Microinjury
			Skeletal muscle	Damage
				Microdamage
	Quantitative magnetic resonance imaging			
	QMRI			

After screening the titles and abstracts, 5,712 articles were deemed irrelevant. The remaining articles underwent a full-text review, which resulted in the inclusion of 21 articles for analysis^1^. The inclusion criteria were defined as follows: studies must focus on marathon-related muscle injuries and utilize quantitative magnetic resonance imaging techniques. Studies that did not meet these criteria or were not published in peer-reviewed journals were excluded.

## 3 Results and discussion

### 3.1 General findings

In studies assessing lower extremity muscle microtrauma after marathon exercise, quantitative MRI techniques have now evolved from single-modality applications to advanced multimodal assessment methods. Single-modality quantitative MRI techniques include T2 mapping, Diffusion-weighted MRI (dMRI), Magnetic Resonance Spectroscopy (MRS) and Chemical Exchange Saturation Transfer (CEST). Multi-modal quantitative MRI techniques include combining information from different modalities and single-scan multicontrast imaging methods (Synthetic MRI, SyMRI). Their respective advantages, disadvantages, and potential clinical applications are summarized in Table 2.

**Table 2:** Strengths, limitations and clinical potential of different quantitative MRI techniques in the assessment of lower limb muscle injuries after marathon running.

Technique	Strengths	Limitations	Potential applications
T2 mapping	Highly sensitive to changes in overall tissue water content and to intra- and extracellular water redistribution	Lack of standardization, e.g., establishment of normal values for T2 in muscle tissue, image acquisition and analysis processes	Assessing the dynamics of muscle inflammatory edema and difference in site of injury
			Estimated time for individuals to return to play and risk of re-injury
	Can be applied to conventional MRI equipment		
DTI	Observation and tracking of fiber bundle orientation and microstructure within the muscle	Difficulty balancing signal-to-noise ratio, resolution and acceptable scan time	Fiber bundle length, pinnation angle, and curvature can be calculated algorithmically to reflect biomechanical properties to a certain extent
	Detection of subclinical changes in lower limb muscles after long-distance running, e.g., minor tears in muscle fibers, abnormal diffusion of water molecules	Lack of multi-center comparisons	
			Assessment of subclinical microinjuries in lower limb muscles induced by different types of marathon exercise
IVIM	No need for intravenous contrast	The effect of marathon exercise on muscle microcirculatory perfusion is unclear	IVIM, based on multiband acceleration technology, greatly reduces scanning time and is expected to be applied in routine clinical monitoring
	Also provides information on perfusion and diffusion in tissues		
		Long scanning time	
MRS	Non-invasive detection of intramyocellular lipid content in muscle, which may have a U-shaped relationship with marathon running intensity	High requirements for spatial homogeneity of the static magnetic field, hardware stability, and scanning technology	Adjustment of exercise or rehabilitation programs based on muscle metabolism to optimize treatment results
		Selectable CEST technology	
Multi-modal MRI techniques	Comprehensive information on muscle structure and function can be provided by combining information from different modalities or applying SyMRI techniques	Combining different modal imaging techniques requires longer scanning times	Multiple sets of quantitative parameters can comprehensively detect microscopic changes in muscles before and after exercise, accurately reflecting the degree of injury and monitoring the recovery process

### 3.2 Single-modality quantitative MRI techniques

#### 3.2.1 T2 mapping

Magnetic resonance T2 mapping is a commonly used method to quantify motor physiologic impairments in lower extremity muscles. The technique obtains multiple T2-weighted images (T2WI) using a series of different echo times (TE) and fitting them with mono-exponential or multi-exponential models to obtain T2 values, and also generates pseudo-color maps for a more objective assessment of the T2 relaxation time of muscle tissue (13). T2 relaxation time is highly sensitive to changes in the overall water content of muscle tissue and to intra- and extracellular water redistribution (14–16). Under normal conditions, T2 values are low due to the orderly arrangement of collagen fibers and the structural integrity of cell membranes in muscle. When the muscle underwent intense exercise, the water content and freedom of skeletal muscle cells increased, resulting in the extension of T2 value. This change reflects microscopic changes in muscle tissue that may occur as a result of pathological conditions such as inflammation and edema (11, 17).

In two recent studies (18, 19) the dynamics of T2 values in the hamstring muscle before and after marathon exercise were quantified by the T2 mapping technique; the results showed a significant prolongation of the muscle's T2 values after exercise compared with those before exercise, suggesting that marathon running may induce inflammatory edema in the hamstring muscle. In another study (10), T2 values of foot muscles before and after 22 college students participated in a full marathon were also analyzed using the T2 mapping technique; the results showed that the T2 values of all extrinsic and some intrinsic foot muscles (plantar flexors) were significantly elevated on day 1 after the race compared to the pre-race period, and that the T2 values were gradually restored to the baseline level by day 8 after the race; moreover, changes in the T2 values of extrinsic foot. In addition, the changes in T2 values of external muscles of the foot (flexor digitorum longus and flexor pollicis longus) between pre-competition and post-competition day 1 were directly related to the corresponding changes in the arch height ratio. This indicates that T2 mapping can accurately reflect the differences in foot muscle injuries caused by full marathon, and the changes in T2 values are reversible, suggesting that micro-injuries of foot muscles can be recovered. Therefore, T2 mapping can be used to quantitatively assess the changes and differences of lower limb muscle inflammatory edema caused by marathon exercise, and may be used to estimate the time to resume marathon participation and the risk of re-injury.

#### 3.2.2 dMRI

dMRI is an imaging technique that reflects microstructural changes by detecting the diffusion properties of water molecules within tissues. It holds significant potential for application in sports medicine, particularly in the assessment of muscle injuries related to marathon running. dMRI encompasses various imaging modalities, with this paper focusing on Diffusion Tensor Imaging (DTI) and Intravoxel Incoherent Motion Imaging (IVIM), discussing in detail their application in the evaluation of lower limb muscle injuries following marathon exercise.

##### 3.2.2.1 DTI

DTI is based on the stochastic Brownian motion of water molecules in living tissues, and a diffusion-sensitive gradient field is applied in multiple directions to quantify the anisotropy of water molecule diffusion, and to observe and track the orientation of fiber bundles and the microstructure within the muscles of the lower extremities. Under normal conditions, the diffusive motion of water molecules in tissues is blocked by intact cell membranes, allowing water molecules to move in the direction of the long axis of the muscle fibers, and the muscle fibers are aligned in an orderly fashion (12). After marathon running, lower limb muscles may show subclinical changes (8), such as minor tears in muscle fibers and disruption of cell membrane integrity, leading to enhanced free diffusion of water molecules and increased free water content between muscle fibers. Subclinical changes of muscle fibers that could not be detected by conventional T2WI sequences can be demonstrated by DTI technique, e.g., minor tears in muscle fibers, abnormal diffusion of water molecules, and are usually manifested as a fractional anisotropy (FA) of the muscle on the image is significantly decreased and mean diffusivity (MD) and apparent diffusion coefficient (ADC) values are increased (20–22). DTI measurements of lower limb muscles can be performed using 1.5T or 3T MRI equipment, and technical adjustments in sequence design (e.g., using b-values between 400 and 600 s/mm² and at least 12 diffusion gradient directions) are usually required to obtain accurate measurements (8, 12, 20).

Furthermore, the available evidence is inconsistent. A recent study of 20 mountain ultra-marathoners found (23) that both T2 and T2* values of the quadriceps were significantly higher after the race compared with before the race and did not return to baseline levels after 48–72 h; this suggests that mountain ultra-marathons may lead to recurrent eccentric contractions and exposure of the quadriceps muscles of the lower limb to high mechanical stress, which in turn lead to quadriceps injuries that are not easy to recover from. In contrast, a study of 17 amateur marathoners participating in a half-marathon showed (20) that DTI measurements (thigh muscle group FA values) changed reversibly before, 3 h, and 3 days after exercise, demonstrating that running on a flat and non-gradient surface is less susceptible to lower limb muscle kinematic injuries. Therefore, DTI is promising for detecting subclinical changes in lower limb muscles and monitoring recovery after different marathon runs. However, the use of DTI measurements as markers in the clinical setting remains challenging due to the difficulty of balancing signal-to-noise ratio, resolution, and acceptable scanning time, and also the need for reference values and large samples for prospective studies.

##### 3.2.2.2 IVIM

IVIM is a noninvasive MRI technique that utilizes multiple b-value dMRI to separate and quantify “true diffusion” and “pseudo-diffusion” information of water molecule movement and microcirculatory perfusion in tissues (24). “Based on a biexponential model, perfusion and diffusion components can be determined simultaneously in lower limb muscle tissue (25).” A small prospective study involving 16 healthy recreational marathon runners (18) showed that the IVIM microvascular perfusion fraction f of the popliteus muscle was significantly higher 2–3 h after participation in a half-marathon compared with the pre-run period, suggesting an increase in blood perfusion. However, another study of 109 athletes with acute hamstring injuries showed (26) that a modified DTI sequence scan of the IVIM model of the bilateral thighs, performed 7 days after injury, revealed significant differences in all IVIM-DTI diffusion parameters (e.g., FA, MD) between the injured side and the contralateral healthy muscle, with the exception of the perfusion fraction, f. To enable routine clinical monitoring of hamstring injuries, this study also proposed an IVIM-DTI method using a multiband acceleration technique, which reduced the scanning time from 11 min 08 s to 3 min 40 s without compromising the sensitivity of the quantitative outcome parameters to hamstring injuries. Thus, the effect of marathon exercise on lower limb muscle microcirculatory perfusion is unclear. Future studies need to analyze larger longitudinal sample sizes by IVIM or IVIM-DTI sequences with multiband acceleration techniques to make routine clinical monitoring of marathon exercise-induced lower extremity microinjuries possible.

#### 3.2.3 MRS and CEST

MRS is an examination method based on the principle of MRI, which identifies and quantifies different chemical substances and their contents by utilizing small differences in the resonance frequencies of atomic nuclei in different chemical environments within the body to distinguish between different chemical shifts. This technology allows for non-invasive detection of energy metabolism in living muscle tissue. Currently, MRS studies of the effects of marathon running on lower limb muscle metabolism have focused on both ¹H-MRS and ³¹P-MRS, which can be measured using 1.5T, 3T, or mobile MRI equipment (27–29). But there is little evidence in the literature that MRS Images muscle damage after marathon running. A small prospective study (30) involving 12 male runners with regular endurance training examined the effects of marathons of varying intensities and durations on muscle; the study measured intramyocellular lipid (IMCL) content in the tibialis anterior and soleus muscles before and after exercise with the ¹H-MRS, and the results showed that the content of IMCL in the tibialis anterior muscle and soleus muscle decreased significantly with the increase of the duration of moderate intensity marathon running. However, when the intensity was higher and the duration was similar, the IMCL content did not decrease significantly. In another more interesting study (31), IMCL content in the tibialis anterior muscle was measured pre- and mid-race in 22 regular endurance runners participating in a multi-stage ultra-marathon using a mobile ¹H-MRS, and the results showed a significant increase in IMCL values compared to the pre-run period during higher-intensity, longer-duration aerobic exercise. Thus, in regular endurance runners, lower limb muscle IMCL content may have a U-shaped relationship with intensity and duration of marathon running. IMCL content at different intensities and durations of marathon running is critical in explaining the role of fatty acids as a source of energy within skeletal muscle and in relation to insulin sensitivity. However, the high requirements of MRS for spatial homogeneity of static magnetic field, hardware stability, and complex scanning techniques, especially ³¹P-MRS, which requires additional hardware equipment and software for transmitting/receiving at the appropriate resonance frequencies, limit its clinical application in studying the effects of marathon running on lower limb muscles. In addition, ¹H-MRS and ³¹P-MRS cannot measure free creatine (Cr) in energy metabolism (32), whereas the emerging CEST technology can measure changes in free Cr (32, 33), pH values (34), and lactate concentrations (35) in human leg muscles before and after mild to moderate exercise. CEST is a non-invasive, high-resolution biochemical imaging tool that uses specific pulses to saturate hydrogen protons in macromolecules and then exchanges the saturated signal with hydrogen protons in surrounding free water. This allows for measuring the difference in free water signals to indirectly reflect macromolecule concentrations. Therefore, future research could consider using CEST technology to monitor the impact of marathons on lower limb muscle metabolites, which may provide new insights into muscle microtrauma.

### 3.3 Multi-modal quantitative MRI techniques

At present, in order to fully analyze the imaging mechanism of the lower limb muscle microtrauma caused by marathon, multi-modal quantitative MRI technology is adopted in the research. Through the combination of different modes or the multi-contrast imaging of a single scan, the muscle structure and function are comprehensively analyzed from multiple angles. For example, in a study of 20 extreme mountain ultra-marathon runners (23), the quadriceps muscle was automatically segmented (36) by a T1 3D sequence based on the Dixon algorithm to quantify its volume, and based on this T1 structure image, the proton density fat fraction (PDFF), T2*, and internal magnetic susceptibility (*χ*) have been simultaneously calculated using 3D spoiled gradient echo sequence, and muscle T2 values were quantified by a 2D multiecho T2-weighted spin-echo sequence. Combined multimodal analyses of quadriceps structure, energy reserve status, and cellular water content revealed significant increases in T2 and T2* values and a slight increase in quadriceps volume from 48 to 72 h post-competition, whereas PDFF and *χ* remained relatively stable, suggesting that the multimodal quantification technique may reveal that extreme eccentric loading runs place significant biomechanical stresses on the quadriceps muscle that translate into a pronounced inflammatory burden. However, the combination of different modal techniques tends to add additional scan time and is limited in clinical applications.

SyMRI techniques greatly reduce scan time by allowing multiple sequences of contrast-weighted images to be acquired in a single image acquisition. For example, in a study of 24 amateur marathon runners (37), knee joints were scanned using SyMRI using a multi-delay multi-echo sequence. Conventional contrast weighted images such as T1WI, T2WI, proton density weighted imaging (PDWI) and short time inversion recovery (STIR), as well as quantitative maps T1, T2 and corresponding relaxation rates R₁ (1/T1), R₂ (1/T2) and proton density (PD) were obtained within 8 min and 5 s. The results showed that T1, T2, and PD values were elevated in the periprosthetic muscle subregion of the knee joint in most subjects compared to the pre-race period within 48 h after the marathon, and decreased after 1 month of rest (37). In conclusion, the various quantitative parameters obtained from the SyMRI technique effectively detect microscopic changes in muscles before and after marathon exercise, offering a valuable tool for the future monitoring of muscle-related diseases.

## 4 Conclusion

The microtrauma caused by marathon exercise to lower limb muscles is manifested in imaging as a diversity of microstructural and functional changes. Quantitative single-modality MRI techniques such as T2 mapping, DTI, IVIM, MRS and CEST are effective in capturing changes in inflammatory edema, subclinical alterations, microcirculatory perfusion, and energy metabolism in muscles, and provide a powerful tool for quantitative assessment of sports injuries. However, the limitations of single-modal techniques may not fully reveal the whole picture of sports injuries in some cases. By combining information of different modes or SyMRI technology, multi-modal quantitative MRI technology can more comprehensively analyze the structural and functional damage of lower limb muscles caused by marathon exercise, especially in extreme mountain ultra-marathon high-intensity exercise, providing important biomechanical and inflammatory burden indicators. However, a major problem is the lack of standardization in the process of clinical conversion of both single-modal and multimodal MRI techniques. Therefore, prospective longitudinal studies with large sample sizes are necessary to determine standardized processes for these technologies.

Future studies should further explore the potential application of quantitative MRI to observe the longitudinal trends of marathon runners after lower extremity muscle injury by plotting curves through the time axis (*x*-axis) and quantitative measurements (e.g., T2, T2* values, diffusion, perfusion parameters, and metabolite content) on the *y*-axis, in an attempt to determine the threshold for safe return to play; such quantitative thresholds would help clinicians, compared with traditional MRI assessments, to physicians to more accurately assess marathoners’ return-to-play time and risk of reinjury (38). In addition, prospective longitudinal studies should expand the sample size to cover different types of marathon events and diverse athlete populations to validate the accuracy and reliability of these techniques in a wider range of applications. Meanwhile, attention should be paid to the potential application of multimodal techniques such as SyMRI-based techniques, exploring their advantages in rapid scanning and comprehensive analysis, aiming at efficient and accurate sports injury monitoring and assessment, and providing a more reliable basis for rehabilitation and re-injury risk prediction in athletes or active individuals.

## Footnotes

^1^The included articles are marked by an asterisk (*) in the reference list.
